# Semantic-Aware Co-Parallel Network for Cross-Scene Hyperspectral Image Classification

**DOI:** 10.3390/s25216688

**Published:** 2025-11-01

**Authors:** Xiaohui Li, Chenyang Jin, Yuntao Tang, Kai Xing, Xiaodong Yu

**Affiliations:** School of Computer Science and Information Engineering, Harbin Normal University, Harbin 150025, China; lxhhrb@hrbnu.edu.cn (X.L.); jinchenyang@stu.hrbnu.edu.cn (C.J.); 2024300691@stu.hrbnu.edu.cn (Y.T.)

**Keywords:** hyperspectral image classification, domain generalization, CNN, multimodal, cross-scene

## Abstract

Cross-scene classification of hyperspectral images poses significant challenges due to the lack of a priori knowledge and the differences in data distribution across scenes. While traditional studies have had limited use of a priori knowledge from other modalities, recent advancements in pre-trained large-scale language-vision models have shown strong performance on various downstream tasks, highlighting the potential of cross-modal assisted learning. In this paper, we propose a Semantic-aware Collaborative Parallel Network (SCPNet) to mitigate the impact of data distribution differences by incorporating linguistic modalities to assist in learning cross-domain invariant representations of hyperspectral images. SCPNet uses a parallel architecture consisting of a spatial–spectral feature extraction module and a multiscale feature extraction module, designed to capture rich image information during the feature extraction phase. The extracted features are then mapped into an optimized semantic space, where improved supervised contrastive learning clusters image features from the same category together while separating those from different categories. Semantic space bridges the gap between visual and linguistic modalities, enabling the model to mine cross-domain invariant representations from the linguistic modality. Experimental results demonstrate that SCPNet significantly outperforms existing methods on three publicly available datasets, confirming its effectiveness for cross-scene hyperspectral image classification tasks.

## 1. Introduction

Deep learning techniques have made significant advancements in hyperspectral image (HSI) classification, with convolutional neural networks (CNNs) being a key component [1,2,3]. CNNs are used to extract and analyze the rich information embedded in hyperspectral images and have been successfully applied to various fields, including environmental monitoring [4], medical imaging [5], agriculture [6], and geological exploration [7]. These networks have demonstrated outstanding performance across these domains. In the context of cross-scene hyperspectral classification, however, training and testing samples often originate from different scenes with distinct spectral and spatial characteristics, resulting in domain shift or distribution mismatch. Such discrepancies may stem from variations in environmental conditions—such as lighting, weather, or geographic location—as well as differences in sensors. Consequently, models trained in one scene may struggle to generalize to others, which can significantly degrade performance. Addressing this challenge requires the development of methods that can effectively learn domain-invariant features and improve model generalization across diverse scenes.

Excitingly, transfer learning has emerged as a powerful approach to address this challenge, with domain adaptation (DA) being the most widely used method for cross-scene classification [8]. A common DA technique is feature alignment [9], which aims to align source and target domain samples into a shared feature space through a mapping, ensuring consistency across different domains [10]. For instance, Long et al. [11] proposed a network, which employs maximum mean discrepancy to reduce domain differences. Additionally, Ganin et al. [12] pioneered the use of adversarial techniques for DA with the development of the domain adversarial neural network. It trains the network to extract similar features from both the source and target domains, thus facilitating domain alignment. These methods focus on aligning features between domains to improve cross-scene classification performance.

During training, DA utilizes data samples and labels from the source domain, along with data samples from the target domain. In recent years, domain generalization (DG) has emerged as a more challenging task than DA. Unlike DA, where data samples and labels from the source domain are used while target domain samples are visible, DG aims to train a model on one or more related domains while ensuring that the target domain remains unseen during training [13]. The goal of DG is to achieve effective generalization to the target domain. To address this, Jin et al. [14] introduced a framework for feature alignment and recovery, which aligns cross-domain features using aligned moments and encourages the separation of relevant from irrelevant features. Meanwhile, Lu et al. [15] proposed the first classification of domain-invariant features into two types: internally invariant features and mutually invariant features, thereby enabling a more comprehensive and diverse extraction of domain-invariant features.

Current deep generalization modeling methods typically focus on manipulating data, learning representations, and developing learning strategies. Among these, representation learning has emerged as the dominant approach [16]. In image classification, the focus is on training models to reduce the representation differences across one or more source domains. Research in multimodal learning has demonstrated that linguistic features can significantly enhance the learning of visual representations [17,18,19], yet this has not been explored in the context of DG. Furthermore, HSI lacks textual descriptive information that can directly reflect feature categories, and the challenge of effectively aligning text and image data remains an open question. To address this, we propose the construction of a semantic space for text-image alignment and optimize supervised contrastive loss to achieve our goal.

This paper proposes a solution to the challenge: a Semantic-aware Collaborative Parallel Network (SCPNet). The network is a multimodal framework that incorporates prior textual knowledge for each category to construct a semantic space. Within this space, features extracted by the spatial–spectral feature extraction module and the multiscale feature extraction module undergo optimized alignment, reducing cross-domain differences. The main contributions of this work are summarized below:

1. We propose a multimodal network for cross-scene hyperspectral image classification, where image features are aligned by category in semantic space to enhance inter-class separability and learn cross-domain invariant representations.
2. We extract rich image features through the collaborative efforts of the spatial–spectral feature extraction module and the multiscale feature extraction module. Compared to using a single module, our parallel network achieves superior feature extraction performance.
3. We construct semantic spaces that facilitate information sharing between the two modalities, enabling the learning of cross-domain invariant representations. We also optimize supervised contrastive loss to ensure better tuning of feature distributions by category.
4. Experiments show that our method outperforms all comparison methods, achieving the best classification results across all three datasets and demonstrating strong generalization ability.

## 2. Related Works

### 2.1. CNN in HSI Classification

CNN-based HSI classification algorithms can be categorized into three groups based on their feature extraction methods: spectral feature extraction, spatial feature extraction, and spatial–spectral feature extraction. Spectral feature extraction typically uses 1D-CNN, which extracts deep spectral features continuously during the convolution process. Chen et al. [20] achieved excellent results at the time using 1D-CNN to extract spectral information from individual pixels. Incorporating spatial information is crucial for hyperspectral image classification, and 2D-CNN is effective in capturing hidden spatial patterns within the image. Song et al. [21] introduced DFFN, which uses PCA for dimensionality reduction and deeply fuses spatial features. DSGSF employs both channel and spatial attention mechanisms to extract and fuse spectral and spatial features, respectively, thereby enhancing the learning of comprehensive global spatial features [22]. Spatial–spectral features are often extracted using 3D-CNN, which offers better feature representation, though it requires more computational resources. Ahmad et al. [23] applied 3D convolution to generate 3D feature maps over multiple contiguous spectral bands. Zhou et al. [24] combined 3D-CNN with feature pyramids to propose SSPN, which captures various spatial–spectral features. More recently, hybrid approaches combining 2D and 3D convolutions for feature extraction, such as CO-PCN [25] and 3D-2D-SSHDR [26], have also been explored. In fact, dual-stream architectures have been widely adopted, such as SimPoolFormer proposed by Roy et al. [27], which has made new progress in hyperspectral image classification and further demonstrated the effectiveness of the dual-stream design in feature modeling. This paper investigates the effectiveness of using convolution for different purposes in two parallel branches of a network for collaborative feature extraction.

### 2.2. Domain Generalization

DG presents a greater challenge compared to DA due to the inclusion of target domain data during training in DA, which is not the case in DG. An approach to address the DG problem is to augment and diversify the source domain data by adding out-of-domain samples, which are then used in training alongside the source domain samples. Zunair et al. [28] proposed MSL, feeding both pre-masking and post-masking images into two identical encoders simultaneously, and enhancing the model’s generalization performance through label consistency. Additionally, variational autoencoders [29] and generative adversarial networks [30] can be employed to generate data samples that aid model generalization. Representation learning is currently a key research direction for DG problems. Hu et al. [31] introduced multidomain discriminant analysis, which maps all source domain data to a high-dimensional feature space, achieving desirable properties. Krueger et al. [32] proposed variance of training risks (VREx) to improve robustness against domain distribution bias. Furthermore, domain adversarial learning [33] can reduce cross-domain variance, and all these methods aim to promote domain-invariant learning.

Another category of research focuses on improving generalization ability through specific learning strategies. For instance, Sagawa et al. [34] significantly improved the accuracy of worst-grouping by combining group distributionally robust optimization (GroupDRO) with stronger regularization. Dong et al. [35] enhances medical image segmentation models by providing additional source domain a priori knowledge through the construction of a memory bank. We draw inspiration from representation learning and aim to learn domain-invariant representations through semantic spaces. This approach involves not only relying on traditional training datasets but also incorporating rich linguistic modalities to enhance the model’s generalization ability. By doing so, we expect the learned representations to be better suited for adaptation to different scenarios.

### 2.3. Vision-Language Model

The learning of visual language models involves the analysis and fusion of heterogeneous data from two distinct sources: text and images. These models can be categorized into single-stream and dual-stream architectures based on whether the data undergoes feature extraction before fusion. Single-stream architectures typically treat textual and image information as equally important, feeding both directly into the fusion module for pre-training, as seen in models like OSCAR [36], UNITER [37], and SimVLM [38]. The advantage of dual-stream architectures lies in their flexibility, as different encoders can be used to process each modality’s data before fusion, allowing for more efficient integration. CLIP [39] employs this approach, utilizing contrastive learning. Similarly, DALSCLIP [40] learns better domain-invariant representations from both images and textual cues. Coca [41], among others, aims to bring positive feature pairs closer together while pushing negative pairs farther apart to extract meaningful representations. This approach not only enhances the performance of traditional visual tasks but also opens new possibilities for processing large-scale remote sensing datasets. By effectively fusing image and text information, visual language models can transcend domain and task boundaries, supporting more complex scene understanding. These models have been applied to key remote sensing tasks, including image retrieval based on text [42,43], image generation from text [44,45], scene categorization [46,47], and object detection [48,49].

## 3. Proposed Method

**Figure 1:**
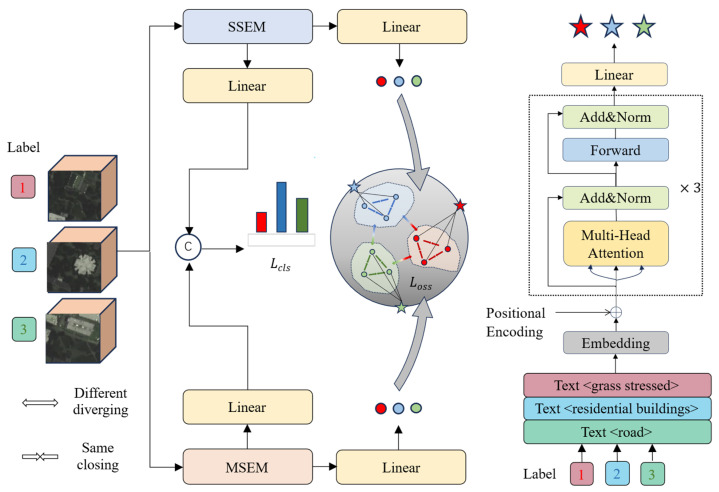
Diagram of SCPnet’s general training framework.

Our proposed method consists of two main components: the image encoder and the optimized semantic space. Figure 1 illustrates the overall training framework of the method. In the image encoder section, we combine a spatial–spectral feature extraction module (SSEM) and a multiscale feature extraction module (MSEM) to comprehensively extract spatial and spectral features from the image. The extracted features are then mapped to the optimized semantic space (OSS), which can enhance the model’s domain generalization capability.

### 3.1. Image Encoder

SCPNet’s image encoder follows a parallel network framework. Within this framework, the SSEM, based on 3D convolution, and the MSEM, based on a residual pyramid structure, form two branches of the same encoder that work synergistically for hyperspectral image feature extraction. The SSEM extracts spatial–spectral features by leveraging 3D information, while the MSEM captures multilevel, detail-rich features through the multiscale residual pyramid structure. The combined efforts of these two modules effectively capture both spatial and spectral information in hyperspectral images, thereby enhancing the comprehensiveness and accuracy of feature representation.

Three-dimensional convolution has become increasingly popular in HSI classification. Such networks stack multiple convolution–pooling layers to form a deep architecture, where the input 3D feature maps are convolved with 3D filters and passed through nonlinear activations to produce output feature maps. The process of 3D convolution can be described as follows:

(1) $$f_{ij}^{xyz}=\sum_{h=0}^{H_{i-1}}\sum_{v=0}^{V_{i-1}}\sum_{c=0}^{C_{i-1}}\left(W_{ijk}^{hvc}f_{\left(i-1\right)k}^{\left(x+h\right)\left(y+v\right)\left(z+c\right)}+b_{ij}\right),$$

where $f_{ij}^{xyz}$ denotes the value of the neuron at the jth feature sample $\left(x,y,z\right)$ in the ith layer of the network. h, v, and c are the length, width, and the number of channels of the 3D convolution, respectively. y denotes the weight of the (i−1) th feature map in the $\left(h,v,c\right)$ convolution kernel, and $b_{ij}$ denotes the network bias.

**Figure 2:**
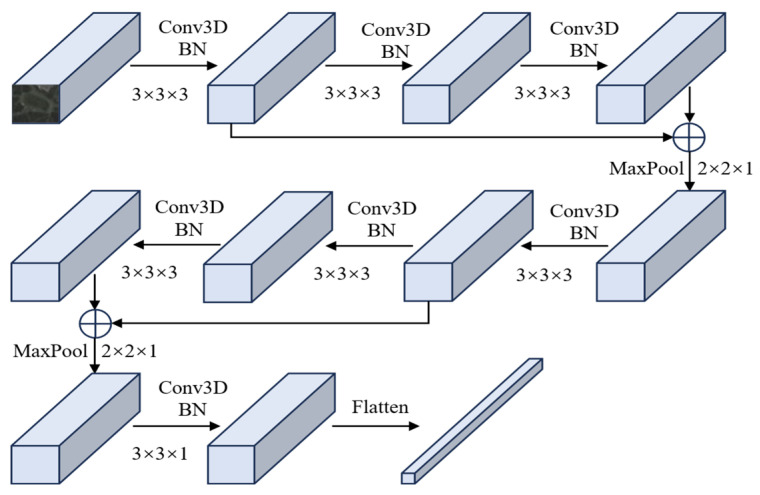
Flowchart of the SSEM of the image encoder.

In the SSEM, as shown in Figure 2, spatial–spectral features are extracted using two 3D convolutional residual blocks, two max-pooling layers, and a 3D convolution. Each residual block contains two Conv3D-BN3D-ReLU modules and a Conv3D layer, with the outputs of the first and last modules combined via residual concatenation.

In the MSEM, the graph pyramid bottleneck residual structure is utilized. Unlike conventional convolutional bottleneck cells, where the depth remains constant across layers, the output of the pyramidal bottleneck residual cell $B_{i}^{j}$ increases in size as the layers progress.

**Figure 3:**
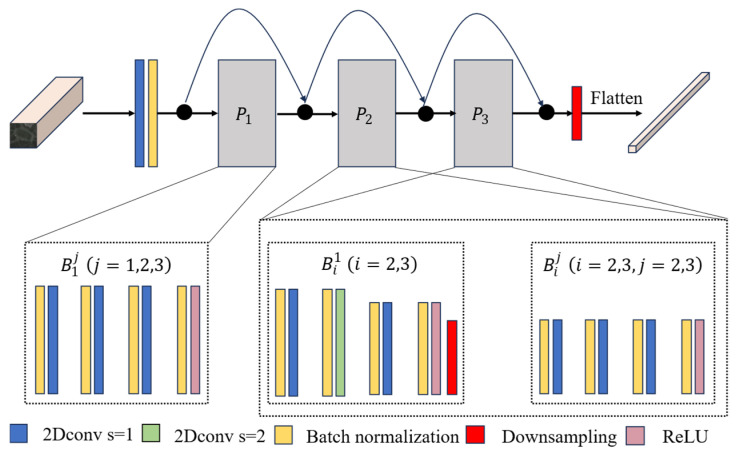
Flowchart of the MSEM of the image encoder.

The network flow of the MSEM is shown in Figure 3. It starts with a Conv2d-BN2d block with a convolutional kernel size of 3 and then goes through three pyramidal residual modules $P_{i}\left(i=1,\;2,\;3\right)$. Where $P_{1}$ contains three residual modules $B_{1}^{j}\left(j=1,\;2,\;3\right)$ of the same structure, in each residual module, three BN2d-Conv2d blocks and one BN2d-ReLU block, with a kernel size of 7 for the second convolutional layer, and a kernel size of 3 for the remaining kernels. Every first residual module $B_{i}^{1}\left(i=2,\;3\right)$ in $P_{2}$ and $P_{3}$ in which the second convolutional layer has a kernel size of 8 and a step size of 2, and the rest is like that in $P_{1}$. At the end of the $B_{i}^{1}\left(i=2,\;3\right)$ unit, a downsampling layer is added to reduce the data space. The structure of the last two residual units, $B_{i}^{j}\left(i=2,\;3,\;j=2,\;3\right)$ in $P_{2}$ and $P_{3}$, is exactly similar to that of $P_{1}$. The network is also connected to an average pooling layer at the end for down sampling to diminish data variance and extract low-level features of the spatial neighborhood for input into the subsequent layer. To systematically augment the depth of the feature map in each layer, set the initial value ${\;N}_{i}^{j}$ to A when i=1 and j = 1. In all other cases,${\;N}_{i}^{j}$ is computed according to the following recursive relationship:

(2) $$N_{i}^{j}=N_{i}^{\left(j-1\right)}+\frac{\alpha }{N^{\left(net\right)}},$$

where A is the initial number of channels, $N_{i}^{\left(j\right)}$ is the depth of the jth unit in the ith pyramid residual module, and $N^{\left(net\right)}$ is the total number of residual units in the entire network. At this point the depth of each layer of the feature map depends on A and α.

The features extracted from SSEM and MSEM are concatenated, and the classification probabilities are produced through the classification header. Subsequently, the cross entropy is calculated using the ground truth. The classification loss of the network is defined as follows:

(3) $$L_{cls}\left(X,Y\right)=-\frac{1}{N}\sum_{i=1}\sum_{c}y_{i}^{c}\log p_{i}^{c},$$

where $y_{i}$ denotes the one-hot vector representing the ground-truth category of sample $x_{i}$, and $p_{i}$ is the predicted probability.

### 3.2. Optimized Semantic Space

Given a source domain $D_{src}={\left\{\left(x_{src}^{i},y_{src}^{i}\right)\right\}}_{1}^{n_{src}}$ and a target domain $D_{tar}={\left\{\left(x_{tar}^{i},y_{tar}^{i}\right)\right\}}_{1}^{n_{tar}}$, where x is the data and y is the label, the data distribution in the $D_{src}$ is not the same as $D_{tar}$, i.e., $X_{src}\neq X_{tar}$. Our goal is to learn two robust and generalized functions $f_{ssem},\;f_{msem}$ from the $D_{src}$ that can be tested on the unseen $D_{tar}$ with the smallest possible prediction error:

(4) $$\underset{f}{\min}E_{\left(X,Y\right)\in D_{tar}}\left[L_{cls}\left(f_{ssem}\left(X\right),Y\right)+L_{cls}\left(f_{msem}\left(X\right),Y\right)\right],$$

where E is the expectation.

We introduce a semantic space that enables the learning of cross-domain invariant representations by learning a cross-domain shared representation space in which images from the source and target domains can be similarly represented. Establishing this semantic space requires introducing prior knowledge, which is processed by the transformer to obtain text features. For the knowledge base $K=\left\{t_{1},t_{2},\ldots ,\;t_{n_{src}}\right\}$, each textual prior knowledge $t_{i}$ corresponds one-to-one to a label $y_{src}^{i}$. The textual prior knowledge describes the spatial relations and imaging features of the category to which the label corresponds, thus providing each label with semantic information related to its category. Given a label $y_{src}^{i}$, we can find the text $t_{i}$ corresponding to it by a simple mapping relation M:

(5) $$t_{i}=M\left(y_{src}^{i}\right),\;where\;M:Y\to T.$$

Calculating the above for the labels of a batch of data eventually results in the prior knowledge T for that batch. Next, we need to encode the text, convert it to a text embedding, and add a positional encoding. The formula is as follows:

(6) $$T=Embed\left[BPE\left(T\right)\right]+PE,$$

where BPE is the lowercase byte pair encoding with a vocabulary size of 49,152, Embed is the token embedding operation, and PE is positional encoding.

After that, T is extracted by transformer architecture to obtain text features. Like a traditional transformer, we utilize a multi-head attention mechanism. The tokenized text $T\in R^{S\times B\times D}$ is divided into h parts, each of which is set to $\;{\hat{T}}_{\alpha }\in R^{S\times B\times \left(D/h\right)}$, α=1, 2,…,h. Where B is the size of batch, S represents the maximum sentence length, while D denotes the size of the embedding dimension, h is the number of headers. ${\hat{T}}_{\alpha }$ is linearly transformed to obtain Q, K, and V. The following formula describes the transformation:

(7) $$\left\{Q_{\alpha },K_{\alpha },V_{\alpha }\right\}\in R^{3\times S\times B\times \left(D/h\right)}=\left\{W_{q},W_{k},W_{v}\right\}\cdot {\hat{T}}_{\alpha },$$

where $W_{q}$, $W_{k}$ and $W_{v}$ are learnable matrices.

Then, the attention weight matrix of each head is computed, and the attention matrices of all heads are combined with the formula defined as follows:

(8) $$A_{\alpha }\in R^{S\times B\times \left(D/h\right)}=Softmax\left(\frac{Q_{\alpha }\cdot K_{a}^{T}}{\sqrt{d}}\right)\cdot V_{\alpha },$$

(9) $$Att\left(T\right)\in R^{S\times B\times D}=Concat\left(A_{1},A_{2},\cdots ,A_{h}\right),$$

where Softmax is used to do SoftMax operation on each sequence of data and Concat combines the attention matrix of each head into a single attention matrix.

The equation for the overall transformer process is simplified as follows:

(10) $$T\in R^{S\times B\times D}=LN1\left(Att\left(T\right)+T\right),$$

(11) $$T\in R^{S\times B\times D}=LN2\left(FFN\left(T\right)+T\right),$$

where LN1 and LN2 are layer normalization operations and FFN is a feed forward neural network.

The described process only involves one layer, and the number of iterations is determined by the number of layers. The transformer consists of three layers, eight headers, and a width of 512. To have a better initial state when the text encoder starts training, the pre-training parameters of ViT-B-32 are used. The final feature $T\in R^{B\times D}$ obtained is the maximum value for each text sequence. Subsequently, we use textual features to build a semantic space representation $Z_{oss}$ and project image features into the semantic space. The formula representation is as follows:

(12) $$\;Z_{oss}=T\cdot W_{oss}+B_{oss},$$

(13) $$Z_{ssem}=Linear\left[f_{ssem}\left(X\right)\right],$$

(14) $$Z_{msem}=Linear\left[f_{msem}\left(X\right)\right],$$

where $W_{oss}$ is the learnable matrix and $B_{oss}$ is the bias term. The linear layer is responsible for mapping the image features into semantic space. Currently, the semantic space and image feature data dimensions are consistent, which is convenient for subsequent operations.

If the model exhibits strong class separability and cross-domain invariance, the visual feature is expected to be located closer in the semantic space to the corresponding linguistic feature associated with its ground-truth label. The following equation describes the calculation process for this similarity:

(15) $$Sim\left(z_{i},z_{j}\right)=\frac{z_{i}^{T}z_{j}}{\left\|z_{i}\right\|\left\|z_{j}\right\|},$$

where $z_{i}$ and $z_{j}$ belong to features of two different modalities. Based on this similarity, the supervised contrastive loss formula can be described as follows:

(16) $$L_{scl}\left(z_{i},z_{j}\right)=-\sum_{i=0}^{N}\frac{1}{\left|P\left(i\right)\right|}\sum_{p\in P\left(i\right)}\log\frac{exp\left(Sim\left(z_{i},z_{j}^{p}\right)\right)}{\sum_{n\in N\left(i\right)}exp\left(Sim\left(z_{i},z_{j}^{n}\right)\right)},$$

where $P\left(i\right)$ and $N\left(i\right)$ are the positive and negative sample sets in $z_{j}$; $z_{j}^{p}$ and $z_{j}^{n}$ are one of the positive and negative samples.

Supervised contrastive learning can reduce the distance between similar features, achieved by calculating the similarity between positive and negative samples. To emphasize the critical role of similarity, we propose an optimized similarity calculation method. Specifically, we square Sim to highlight similarity. The optimized contrastive loss can be described as follows:

(17) $$L_{ocl}\left(z_{i},z_{j}\right)=-\sum_{i=0}^{N}\frac{1}{\left|P\left(i\right)\right|}\sum_{p\in P\left(i\right)}\log\frac{exp\left({Sim}^{2}\left(z_{i},z_{j}^{p}\right)\right)}{\sum_{n\in N\left(i\right)}exp\left({Sim}^{2}\left(z_{i},z_{j}^{n}\right)\right)}.$$

We differentiate the $Sim\left(z_{i},z_{j}^{p}\right)$ with respect to the two types of supervised contrastive loss. For simplicity, we omit the summation and obtain the following two equations:

(18) $$\frac{\partial L_{scl}}{\partial Sim\left(z_{i},z_{j}^{p}\right)}=-1+\frac{exp\left(Sim\left(z_{i},z_{j}^{p}\right)\right)}{\sum_{n\in N\left(i\right)}exp\left(Sim\left(z_{i},z_{j}^{n}\right)\right)},$$

(19) $$\frac{\partial L_{ocl}}{\partial Sim\left(z_{i},z_{j}^{p}\right)}=-2Sim\left(z_{i},z_{j}^{p}\right)+\frac{2Sim\left(z_{i},z_{j}^{p}\right)exp\left({Sim}^{2}\left(z_{i},z_{j}^{p}\right)\right)}{\sum_{n\in N\left(i\right)}exp\left({Sim}^{2}\left(z_{i},z_{j}^{n}\right)\right)},$$

where if $Sim\left(z_{i},z_{j}^{p}\right)$ is large (i.e., the positive pairs are already close), the squared term introduces an amplification factor of $2Sim\left(z_{i},z_{j}^{p}\right)$. This ensures that the optimizer still produces a significant pulling effect, thereby avoiding the vanishing-gradient problem of the original supervised contrastive loss when similarities are high and preventing training stagnation. If $Sim\left(z_{i},z_{j}^{p}\right)$ is small (i.e., negative or irrelevant pairs), the squared value ${Sim}^{2}\left(z_{i},z_{j}^{p}\right)$ rapidly decays, and the gradient approaches zero. This mitigates excessive repulsion of irrelevant negative samples. The final loss in optimized semantic space can be described as follows:

(20) $$L_{oss}=\left(1-\alpha \right)L_{ocl}\left(Z_{ssem},Z_{oss}\right)+{\alpha L}_{ocl}\left(Z_{msem},Z_{oss}\right),$$

where the contribution weight α serves to adjust the degree of influence of the image features of the two branches within the optimized semantic space. With this approach, features from the same class of labels are clustered together, while features from different classes of labels are distanced. Meanwhile, the method crosses the modal divide and successfully learns representations from different modalities, enhancing the generalization ability of the model. The overall loss during model training is defined as follows:

(21) $$L=L_{cls}+\lambda L_{oss},$$

where the hyperparameter λ serves to balance the effects of the optimized semantic space on the overall model training.

In the testing phase, features are extracted and classified using SCPNet’s image encoder incorporating SSEM and MSEM. The training and testing process of SCPNet is shown in Algorithm 1.
**Algorithm 1:** Pseudocode of SCPNet1**Training stage:**2**Input**: Source domain samples $S={\left\{X_{i}^{s},Y_{i}\right\}}_{i=1}^{N_{s}}$, total epoch number T, knowledge base K.3**Output**: The parameters $\theta_{SSEM}$*,*$\;\theta_{MSEM}$4**Initialize**:$\;\theta_{SSEM}$*,*$\;\theta_{MSEM}$. Load Pretrain parameters5**Load:** Pretrain parameters $\theta_{Transformer}$
6**For** epoch = 1: T **do**:7** Extract image features and text features**8**  **$Z_{ssem}=SSEM\left(X\right),\;{\;Z}_{msem}=MSEM\left(xX\right)$9**  **T=Transformer(K) through Equations (5)–(11)10** Establish an optimized semantic space and map image features through Equations (12)–(14)**11** For all**$Z_{ssem},\;{\;Z}_{msem}$, T:12   Calculate the loss $L_{cls}$ through Equation (3)13   Calculate the loss $L_{oss}$ through Equations (15)–(20)14   Calculate the total loss L through Equation (21)15** End For**16 Update $\theta_{SSEM}$*,*
$\theta_{MSEM}$*,*
$\theta_{Transformer}$ by gradient descent17**End For**18**Testing stage:**19**Input**: Target domain samples $T={\left\{X_{i}^{t},Y_{i}\right\}}_{i=1}^{N_{t}}$20**Load:** The parameters $\theta_{SSEM}$*,*
$\theta_{MSEM}$21**Extract image features**22**  **$Z_{ssem}=SSEM\left(X\right),\;{\;Z}_{msem}=MSEM\left(X\right)$23**Output:** $\left[Z_{ssem},{\;Z}_{msem}\right]$ (Classification Vector)

## 4. Experiment and Discussion

### 4.1. Description of Datasets

To verify the effectiveness of our approach, we incorporate three publicly available datasets: Houston, Pavia, and Indiana. Each dataset comprises two scenes, which are elaborated upon below.

**Figure 4:**
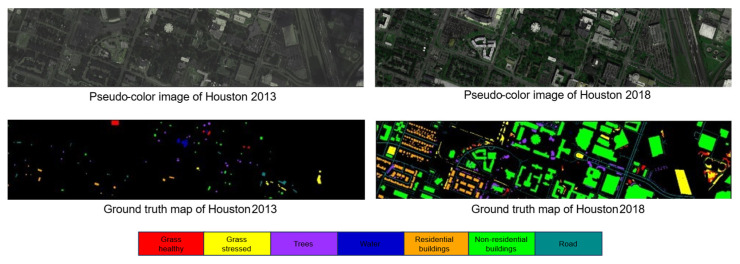
Houston dataset.

The Houston dataset, collected from the University of Houston and its surroundings, includes two scenes: Houston 13 [50] (349 × 1905 pixels, 144 bands) as the source domain and Houston 18 [51] (209 × 955 pixels, 48 bands) as the target domain, both with a spatial resolution of 2.5 m. Acquired at different conditions in 2013 and 2018, the datasets were aligned by selecting the 48 common bands from Houston13 and cropping the overlapping region (209 × 955). Both datasets contain the same seven land cover classes, as detailed in Table 1. The pseudo-color and true value maps for both datasets are shown in Figure 4. Due to the fewer source samples in Houston 13, we applied data augmentation such as random flipping and radiometric enhancement, to expand the training set fourfold.

**Figure 5:**
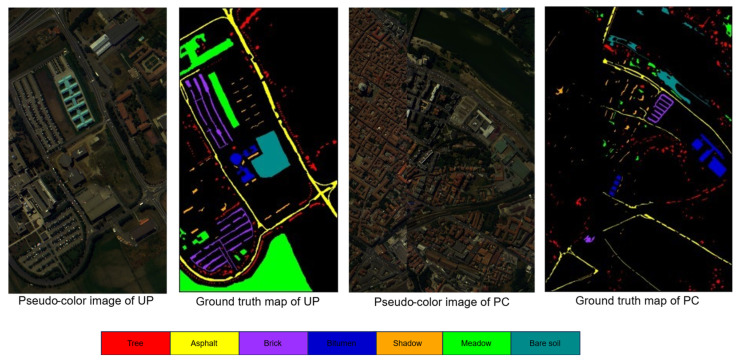
Pavia dataset.

The Pavia dataset was collected from Italy in 2003 using the POSIS sensor [52]. It is divided into two segments: the University of Pavia (UP), which serves as the source domain, containing 610 × 340 image elements and 103 spectral bands, and the Pavia Center (PC), which serves as the target domain, comprising 1096 × 715 image elements and 102 spectral bands. The dataset has a spatial resolution of 1.3 m. By removing the 103rd band, the number of bands in the UP dataset was adjusted to 102 to match the PC dataset. The dataset contains seven feature columns, as detailed in Table 2, and the pseudo-color and true value maps are shown in Figure 5.

**Figure 6:**
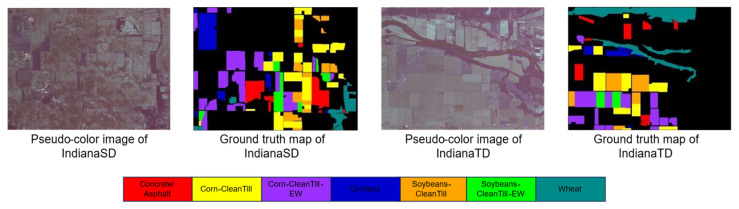
Indiana dataset.

The Indiana dataset was acquired by AVIRIS sensors in the Indian Pine Barrens of northwestern Indiana in 1992. In order to distinguish the source and target domains, the scene is divided into two datasets without overlapping image elements as suggested in the literature [53], which we call IndianaSD and IndianaTD, respectively. The dataset consists of 400 × 300 image elements and 220 spectral bands. The scene includes various land features, such as crops, orchards, and more, with further details provided in Table 3. The pseudo-color and true value maps are shown in Figure 6.

### 4.2. Experiment Setting

All experiments were run in the python 3.6 and pytorch 11.3 environments. The default weight decay for L2 regularization across all modules was set to 1 × 10^−4^. The image encoder and text encoder were optimized by Adam. The CPU was an AMD EPYC 7642 48-core Processor, and the GPU was an RTX 3090 with 24GB of RAM. We created knowledge bases for each of the three datasets, where each category corresponds to a piece of prior knowledge. The knowledge bases for three datasets are shown in Table 4, Table 5 and Table 6.

### 4.3. Parameter Tuning

**Figure 7:**
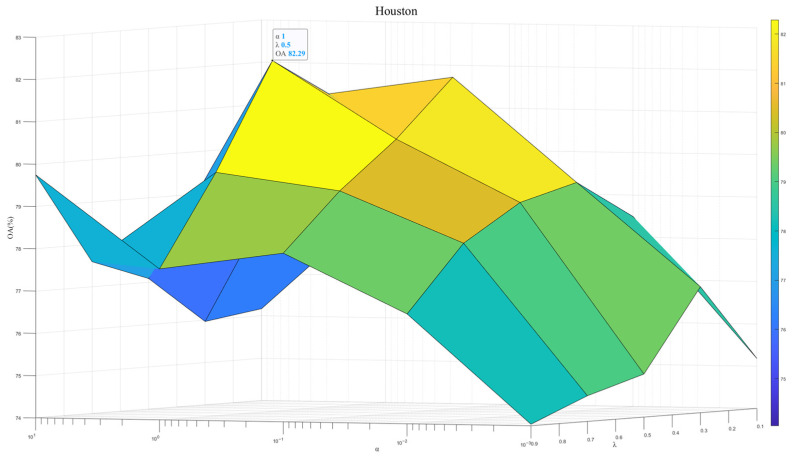
Model performance at different λ and α in Houston dataset.

**Figure 8:**
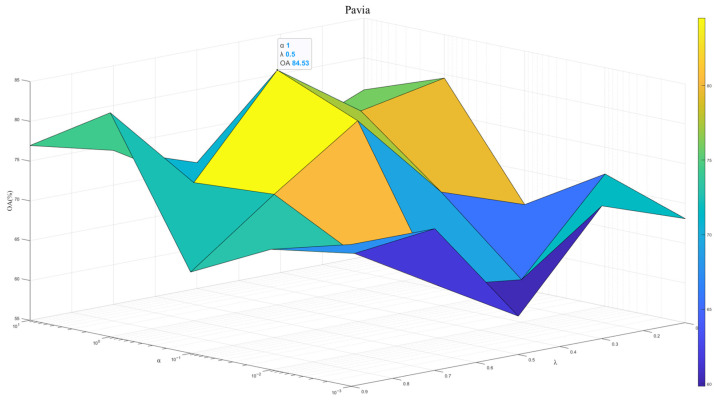
Model performance at different λ and α in Pavia dataset.

**Figure 9:**
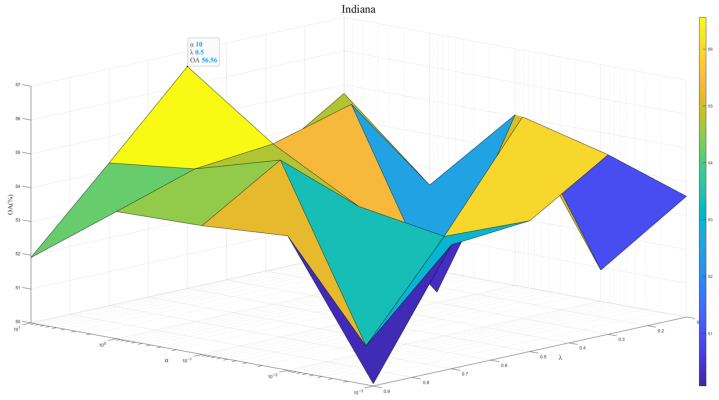
Model performance at different λ and α in Indiana dataset.

To exploit the model potential, we tuned the regularization parameter $\lambda \in \left\{1\times 10^{2},1\times 10^{1},1\times 10^{0},1\times 10^{-1},1\times 10^{-2}\right\}$ and the contribution weight α∈{0.1,0.3,0.5,0.7,0.9}. Figure 7, Figure 8 and Figure 9 show results across three datasets. We recommend λ=0.5, which reflects the same importance of SSEM and MSEM in semantic space. For the Houston and Pavia datasets, the α is set to 1 × 10^−0^, which is effective in preventing overfitting and improving the stability of the model. In contrast, for the Indiana dataset, α of 1 × 10^1^ will help to deal with the problem of large feature differences in this dataset, thus improving the classification performance.

### 4.4. Ablation Study

To verify the effectiveness of our proposed modules, we designed ablation experiments. In the experiments, we separately evaluated the effectiveness of SSEM, MSEM, and OSS. Additionally, SS denotes the use of original supervised contrastive learning. By comparing the experimental results of SS and OSS, we demonstrate the effectiveness of the proposed contrastive learning loss optimization method.

According to Table 7, experimental results show that the proposed SCPNet has superior performance compared to these variants. Specifically, designing SSEM and MSEM as two branches of a parallel network and training them together can give full play to their advantages and achieve complementary advantages. The semantic space and optimized contrastive loss then improve model generalization.

### 4.5. Comparison Experiment

To evaluate cross-domain classification performance, we compared our framework with SDEnet [54], S2ECnet [55], LLURnet [56] and FDGnet [57]. Since the existing cross-scene hyperspectral classification methods are limited, we additionally chose four DG methods for RGB images, namely GroupDRO [34], ANDMask [58], VREx [3], and DIFEX [15], to further validate the effectiveness of our methods. Source domain data and labels were used for training (50% training/50% validation, except 80% training for Houston), while the entire target domain was used for testing. The patch size, batch size, and learning rate were set to 13, 256, and 0.001, respectively. Each method was independently executed ten times with random seeds from 0 to 9, and the results are presented as mean ±95% confidence intervals (CI) to indicate statistical reliability. To measure the accuracy and consistency between the true label and the predicted values, the overall accuracy (OA) and the kappa coefficient (KC) on the target domain were used as the evaluation metrics of the algorithms.

The results are shown in Table 8, Table 9 and Table 10. On the Houston and Pavia datasets, LLURNet achieved the best performance among the compared methods, while our SCPNet further improved OA by 4.22 and 0.86 and KC by 7.16 and 0.96, respectively. On the Indiana dataset, FDGnet performed best among the baselines, but SCPNet still outperformed it by 1.59 in OA and 5.78 in KC. It is also worth noting that although the RGB-based DG methods did not achieve the best results, their competitive performance demonstrates the potential of transferring RGB-based DG strategies to cross-scene hyperspectral classification. In the Indiana dataset, the standard deviation of some categories even exceeded their mean accuracy, indicating large fluctuations and highlighting the challenge of classifying these categories. Our method effectively mitigates this issue and, overall, achieves superior classification performance compared with existing methods.

**Figure 10:**
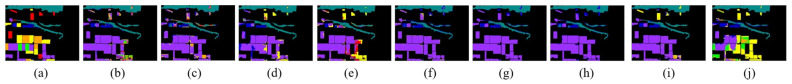
Ground truth and classification maps for IndianaTD. (**a**) GT. (**b**) SDEnet. (**c**) S2ECnet. (**d**) LLURnet. (**a**) GT. (**b**) GroupDRO. (**c**) ANDMask. (**d**) VREx. (**e**) DIFEX. (**f**) SDEnet. (**g**) S2ECnet. (**h**) LLURnet. (**i**) FDGnet. (**j**) Ours.

**Figure 11:**
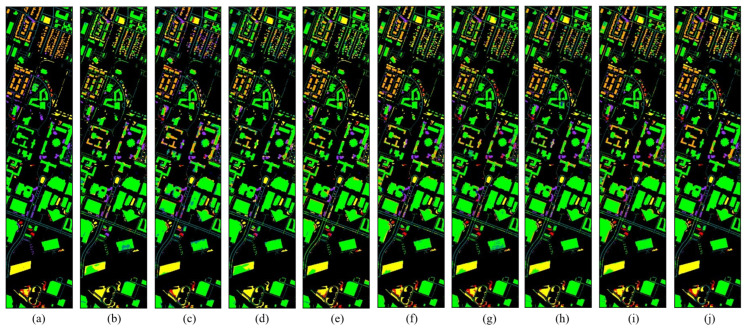
Ground truth and classification maps for Houston 2018. (**a**) GT. (**b**) SDEnet. (**c**) S2ECnet. (**d**) LLURnet. (**a**) GT. (**b**) GroupDRO. (**c**) ANDMask. (**d**) VREx. (**e**) DIFEX. (**f**) SDEnet. (**g**) S2ECnet. (**h**) LLURnet. (**i**) FDGnet. (**j**) Ours.

**Figure 12:**
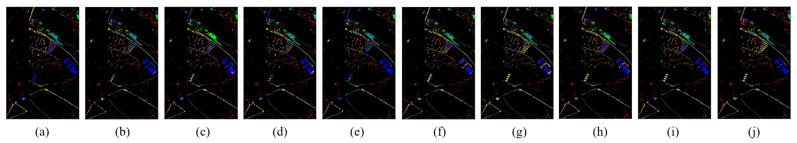
Ground truth and classification maps for Pavia Center. (**a**) GT. (**b**) SDEnet. (**c**) S2ECnet. (**d**) LLURnet. (**a**) GT. (**b**) GroupDRO. (**c**) ANDMask. (**d**) VREx. (**e**) DIFEX. (**f**) SDEnet. (**g**) S2ECnet. (**h**) LLURnet. (**i**) FDGnet. (**j**) Ours.

The classification maps for three datasets are shown in Figure 10, Figure 11 and Figure 12. Each pixel with an unspecified category is used as the background and pixels with categories are predicted for comparison. Each figure contains a truth map and a presentation of the predictions from all models. In Figure 10, we can clearly see that the 2nd and 5th classes are better predicted by SCPnet.

**Figure 13:**
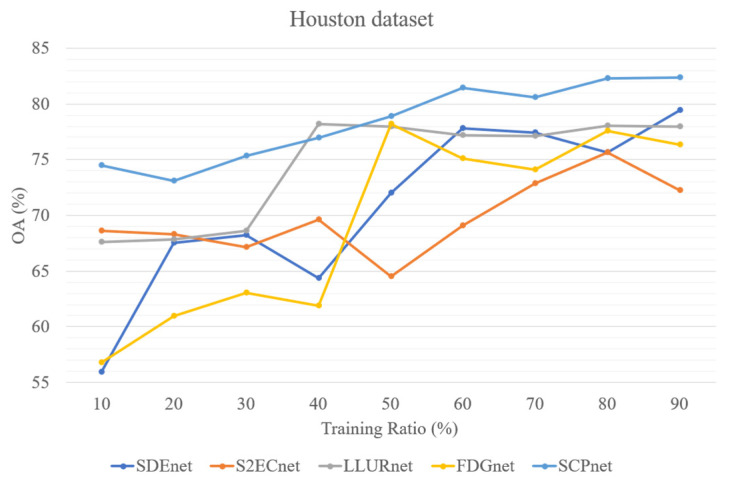
Five methods’ performance at different training ratio on Houston dataset.

**Figure 14:**
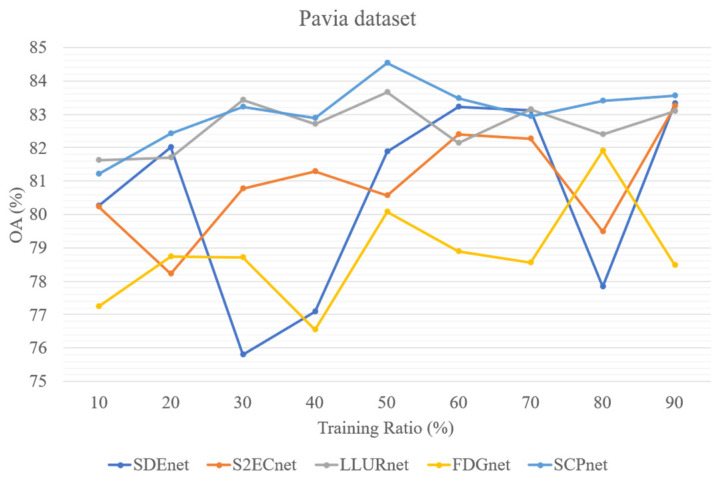
Five methods’ performance at different training ratio on Pavia dataset.

**Figure 15:**
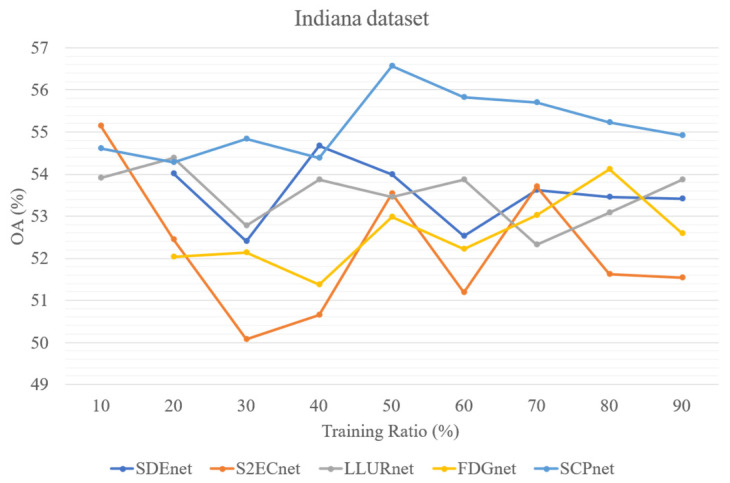
Five methods’ performance at different training ratio on Indiana dataset.

In addition, to test the parameter sensitivity of the parameters, we evaluated four DG methods for cross-scene HSI classification and SCPnet under varying training ratios. Figure 13, Figure 14 and Figure 15 illustrate the results on three datasets, it can be seen that SCPNet consistently exhibits superior generalization across all datasets, demonstrating its robustness to fluctuations in the amount of available training data.

The training time and inference speed are indicative of the computational cost of a model. Accordingly, we measured the training time, testing time, and number of parameters for all models, as summarized in Table 11. SCPNet exhibits a relatively higher computational cost across all three datasets. Specifically, it contains approximately 38.88 M, 47.87 M, and 56.25 M parameters on the Houston, Pavia, and Indiana datasets, respectively, which are considerably larger than those of other approaches. This increase primarily stems from the inclusion of the pretrained language model and the 3D-CNNs used in the SSEM, which enhance feature expressiveness at the expense of additional computation. In terms of training and inference efficiency, SCPNet also requires longer training times (40.27 s, 139.22 s, and 221.39 s, respectively) and testing times (44.75 s, 53.45 s, and 92.31 s, respectively) per batch compared with most competing methods. However, this computational overhead is accompanied by notable improvements in classification accuracy and cross-scene generalization performance.

## 5. Conclusions

We propose the Semantic-aware Collaborative Parallel Network (SCPNet), a domain generalization network, for the cross-scene HSI classification. The key innovation of SCPNet lies in its dual-branch feature extraction modules and the construction of an optimized semantic space, which enable the model to collaboratively extract features and learn domain-invariant representations. Extensive experiments on three benchmark datasets confirm that SCPNet consistently outperforms state-of-the-art baselines, achieving robustness under varying training ratios and across heterogeneous domains. These findings highlight the scientific contribution of integrating semantic-aware learning with co-parallel feature extraction. However, the experiments on computational cost reveal that SCPNet requires substantial computational resources, mainly in terms of longer training and testing time as well as a larger number of model parameters. Nevertheless, these costs are acceptable considering the significant improvements in classification performance achieved by the proposed model. Future research may focus on incorporating prior knowledges from other modalities, or developing lightweight text and image encoders to further reduce the computational burden.

## Figures and Tables

**Table 1:** The name of class and number of samples in Houston dataset.

Class		Number of Samples	
ID	Name	Source Scene (Houston 13)	Target Scene (Houston 18)
1	Grass healthy	345	1353
2	Grass stressed	365	4888
3	Trees	365	2766
4	Water	285	22
5	Residential buildings	319	5347
6	Non-residential buildings	408	32,459
7	Road	443	6365
Total		2530	53,200

**Table 2:** The name of class and number of samples in Pavia dataset.

Class		Number of Samples	
ID	Name	Source Scene (UP)	Target Scene (PC)
1	Tree	3064	7598
2	Asphalt	6631	9248
3	Brick	3682	2685
4	Bitumen	1330	7287
5	Shadow	3947	2863
6	Meadow	18,649	3090
7	Bare soil	5029	6584
Total		39,332	39,335

**Table 3:** The name of class and number of samples in Indiana dataset.

Class		Number of Samples	
ID	Name	Source Scene (IndianaSD)	Target Scene (IndianaTD)
1	Concrete/Asphalt	4867	2942
2	Corn-CleanTill	9822	6029
3	Corn-CleanTill-EW	11,414	7999
4	Orchard	5106	1562
5	Soybeans-CleanTill	4731	4792
6	Soybeans-CleanTill-EW	2996	1638
7	Wheat	3223	10,739
Total		42,159	35,701

**Table 4:** Knowledge base for the Houston dataset.

Class	Name	Prior Knowledge
1	Grass healthy	The grass healthy is lush
2	Grass stressed	The grass stressed by the road appears pale
3	Trees	The trees grow steadily along the road
4	Water	Water appears smooth with a dark blue or black color
5	Residential buildings	Residential buildings arranged neatly
6	Non-residential buildings	Non-residential buildings vary in shape
7	Road	Roads divide buildings into blocks

**Table 5:** Knowledge base for the Pavia dataset.

Class	Name	Prior Knowledge
1	Tree	The trees grow steadily along the road
2	Asphalt	Asphalt is used to pave roads
3	Brick	A brick is a type of construction material
4	Bitumen	Bitumen is a material for building surfaces
5	Shadow	Shadows will appear on the backlight of the building
6	Meadow	Meadow is a land covered with grass
7	Bare soil	No vegetation on the surface of bare soil

**Table 6:** Knowledge base for the Indiana dataset.

Class	Name	Prior Knowledge
1	Concrete/Asphalt	No crops on the surfaces of Concrete or Asphalt
2	Corn-CleanTill	Corn-CleanTill planted with corn
3	Corn-CleanTill-EW	Corn-CleanTill-EW planted with early maturing maize
4	Orchard	The orchard is full of fruit trees
5	Soybeans-CleanTill	Soybeans-CleanTill planted with soybeans
6	Soybeans-CleanTill-EW	Soybeans-CleanTill-EW grows early soybeans
7	Wheat	Wheat is an important food crop

**Table 7:** Ablation experiment results.

SSEM	MSEM	SS	OSS	Houston		Pavia		Indiana	
				OA	KC	OA	KC	OA	KC
✓			✓	75.35	63.05	74.86	69.81	55.27	41.94
	✓		✓	78.32	63.05	72.83	66.93	54.99	41.28
✓	✓			74.72	51.87	71.68	66.42	55.20	41.41
✓	✓	✓		79.81	63.21	78.58	74.33	55.37	41.67
✓	✓		✓	82.29	68.62	84.53	81.37	56.56	44.81

**Table 8:** Comparison of class-specific, OA (%) and KC (κ) of different methods for the target scene Houston 2018.

Class	GroupDRO	ANDMask	VREx	DIFEX	SDEnet	S2ECnet	LLURnet	FDGnet	Ours
1	30.36 ± 7.61	35.02 ± 7.54	33.75 ± 7.85	23.62 ± 6.12	62.94 ± 13.10	**80.34 ± 15.84**	33.06 ± 10.77	58.02 ± 10.98	46.27 ± 12.07
2	71.18 ± 1.65	70.76 ± 2.95	69.87 ± 1.74	68.28 ± 5.27	78.13 ± 5.64	65.09 ± 8.56	69.01 ± 5.72	**77.96 ± 4.38**	67.96 ± 5.87
3	63.98 ± 1.82	64.16 ± 4.38	64.30 ± 3.74	66.41 ± 4.59	57.91 ± 12.53	46.71 ± 3.50	64.90 ± 4.03	67.14 ± 3.98	**68.51 ± 8.10**
4	81.82 ± 5.14	81.82 ± 2.30	78.18 ± 6.25	91.82 ± 8.10	100.00 ± 0.00	100.00 ± 0.00	100.00 ± 0.00	100.00 ± 0.00	**100.00 ± 0.00**
5	50.73 ± 6.54	53.30 ± 7.20	55.58 ± 3.94	54.34 ± 7.60	62.02 ± 3.88	73.62 ± 5.34	67.80 ± 4.01	70.00 ± 10.82	**74.10 ± 3.90**
6	80.92 ± 6.87	78.11 ± 6.46	78.06 ± 4.84	81.87 ± 4.32	84.54 ± 3.15	83.42 ± 1.86	74.25 ± 26.44	84.28 ± 1.36	**94.53 ± 2.33**
7	59.83 ± 6.82	**65.46 ± 4.38**	62.86 ± 6.22	60.13 ± 6.74	50.05 ± 1.97	47.02 ± 4.26	46.63 ± 3.73	57.73 ± 4.59	51.33 ± 6.29
OA	72.30 ± 2.67	71.61 ± 3.05	71.39 ± 1.95	72.97 ± 1.98	75.63 ± 2.07	75.61 ± 1.30	78.07 ± 0.63	77.54 ± 0.37	**82.29 ± 1.01**
KC	55.33 ± 2.28	55.34 ± 3.07	54.65 ± 1.79	55.89 ± 2.22	59.59 ± 2.51	59.96 ± 1.70	61.46 ± 1.65	62.70 ± 1.16	**68.62 ± 2.17**

**Table 9:** Comparison of class-specific, OA (%) and KC (κ) of different methods for the target scene Pavia Center.

Class	GroupDRO	ANDMask	VREx	DIFEX	SDEnet	S2ECnet	LLURnet	FDGnet	Ours
1	79.55 ± 13.27	81.85 ± 4.96	81.51 ± 14.02	82.53 ± 7.87	92.79 ± 2.14	**95.36 ± 2.07**	83.29 ± 5.39	78.24 ± 8.13	9.44 ± 1.46
2	82.50 ± 3.93	76.45 ± 3.12	80.82 ± 2.56	81.55 ± 5.80	84.73 ± 2.20	81.68 ± 6.62	84.48 ± 1.19	76.92 ± 4.71	**87.73 ± 2.95**
3	19.11 ± 11.83	23.07 ± 13.41	21.42 ± 10.04	25.20 ± 21.68	**78.39 ± 6.49**	58.89 ± 25.99	74.67 ± 4.26	68.56 ± 11.45	71.23 ± 6.54
4	73.18 ± 12.26	68.40 ± 16.49	78.13 ± 7.89	74.71 ± 9.97	81.05 ± 1.82	78.44 ± 6.68	84.96 ± 1.13	**86.20 ± 1.09**	82.76 ± 3.04
5	80.00 ± 3.08	80.44 ± 7.73	81.15 ± 4.86	**90.78 ± 4.28**	83.89 ± 5.66	84.48 ± 4.87	86.16 ± 3.93	88.84 ± 1.40	88.95 ± 3.12
6	77.86 ± 4.90	80.82 ± 5.65	76.30 ± 5.31	76.99 ± 3.79	71.42 ± 6.44	61.49 ± 7.38	75.91 ± 4.88	75.26 ± 3.93	**81.49 ± 4.15**
7	74.65 ± 10.06	82.76 ± 7.08	70.79 ± 8.22	69.85 ± 16.45	71.21 ± 3.89	79.58 ± 6.61	87.62 ± 3.75	81.04 ± 8.43	**81.28 ± 7.93**
OA	74.02 ± 3.08	74.04 ± 2.22	74.39 ± 3.13	74.98 ± 3.46	81.89 ± 0.87	80.58 ± 0.30	83.67 ± 0.38	80.09 ± 1.11	**84.53 ± 0.94**
KC	68.64 ± 3.53	68.78 ± 2.54	69.17 ± 3.58	69.98 ± 4.02	78.27 ± 1.02	75.88 ± 1.43	80.41 ± 0.46	76.20 ± 1.29	**81.37 ± 1.19**

**Table 10:** Comparison of class-specific, OA (%) and KC (κ) of different methods for the target scene IndianaTD.

Class	GroupDRO	ANDMask	VREx	DIFEX	SDEnet	S2ECnet	LLURnet	FDGnet	Ours
1	10.29 ± 6.14	11.10 ± 7.54	8.45 ± 6.90	**14.73 ± 15.02**	0.01 ± 0.02	0.63 ± 0.64	0.24 ± 0.39	0.31 ± 0.48	1.43 ± 1.64
2	29.24 ± 18.59	8.47 ± 12.09	4.13 ± 1.12	9.51 ± 14.57	0.82 ± 0.79	2.39 ± 2.52	1.95 ± 2.17	1.78 ± 1.19	**46.39 ± 6.75**
3	68.82 ± 16.44	85.06 ± 9.66	92.21 ± 4.65	86.05 ± 11.96	**94.52 ± 1.82**	92.80 ± 3.84	92.93 ± 3.95	90.79 ± 1.52	44.17 ± 5.46
4	77.89 ± 4.60	70.82 ± 8.46	66.91 ± 18.70	77.55 ± 12.18	83.18 ± 2.93	86.03 ± 2.78	86.89 ± 3.94	**88.98 ± 2.34**	66.07 ± 3.07
5	0.66 ± 0.96	0.10 ± 0.16	0.02 ± 0.02	0.01 ± 0.11	0.01 ± 0.02	0.00 ± 0.00	0.00 ± 0.00	0.00 ± 0.00	**44.03 ± 2.04**
6	0.00 ± 0.00	0.00 ± 0.00	0.00 ± 0.00	0.09 ± 0.14	0.00 ± 0.00	0.00 ± 0.00	0.00 ± 0.00	0.00 ± 0.00	0.00 ± 0.00
7	96.16 ± 1.03	95.40 ± 1.17	96.34 ± 1.44	94.83 ± 1.89	95.96 ± 0.54	94.81 ± 1.72	94.77 ± 1.99	94.96 ± 1.62	**99.42 ± 0.36**
OA	53.65 ± 0.68	53.21 ± 1.24	53.96 ± 0.77	54.48 ± 1.00	54.00 ± 0.26	53.53 ± 0.49	53.46 ± 0.43	54.97 ± 0.41	**56.56 ± 0.46**
KC	40.30 ± 0.57	39.32 ± 1.39	39.86 ± 1.16	41.05 ± 1.44	40.13 ± 0.58	39.55 ± 0.58	39.52 ± 0.55	39.03 ± 0.39	**44.81 ± 0.60**

**Table 11:** Comparison of computational costs across all methods on three datasets.

		GroupDRO	ANDMask	VREx	DIFEX	SDEnet	S2ECnet	LLURnet	FDGnet	Ours
Houston	Train (s)	7.08	10.94	7.59	957.21	16.75	174.87	12.15	22.09	40.27
	Test (s)	7.19	7.16	6.87	7.14	15.16	50.31	10.14	19.99	44.75
	Params (M)	10.80	10.80	10.80	21.78	1.79	1.51	0.55	3.14	38.88
Pavia	Train (s)	9.27	13.97	9.37	1068.02	49.83	356.76	31.32	53.19	139.22
	Test (s)	8.13	7.67	7.92	11.05	22.62	185.69	19.65	24.15	53.45
	Params (M)	10.96	10.96	10.96	22.10	2.41	2.16	0.57	4.04	47.87
Indiana	Train (s)	10.92	15.36	11.12	1172.47	62.56	426.12	51.46	75.30	221.39
	Test (s)	9.91	9.48	9.85	8.81	37.08	267.67	27.76	44.63	92.31
	Params (M)	11.31	11.31	11.31	22.81	3.75	3.58	0.63	6.66	56.25

## Data Availability

Data are contained within the article.
